# Quantification of β-cell insulin secretory function using a graded glucose infusion with C-peptide deconvolution in dysmetabolic, and diabetic cynomolgus monkeys

**DOI:** 10.1186/1758-5996-5-40

**Published:** 2013-07-25

**Authors:** Xiaoli Wang, Barbara C Hansen, Da Shi, Yupeng Fang, Fenglai Du, Bingdi Wang, Yaxiong Michael Chen, Francine M Gregoire, Yi-Xin Jim Wang

**Affiliations:** 1 Cardiovascular and Metabolic Diseases Research, Crown Bioscience Inc., 6 Beijing West Road, Science & Technology Park, Taicang Economic Development Area, Taicang, Jiangsu Province 215400, P.R. China; 2 International Institute of Biomedical Research (IIBR), a Crown Bioscience Company at David H. Murdock Research Institute (DHMRI), Kannapolis, NC, USA; 3 Departments of Internal Medicine and Pediatrics, and Obesity, Diabetes and Aging Research Center, University of South Florida, Tampa, FL, USA

**Keywords:** Insulin secretion rate, C-peptide, Deconvolution, Graded glucose infusion, GGI, Insulin resistance, β-cell failure, Diabetes, Hepatic insulin extraction, Nonhuman primate

## Abstract

**Background:**

Quantitation of β-cell function is critical in better understanding of the dynamic interactions of insulin secretion, clearance and action at different phases in the progression of diabetes. The present study aimed to quantify β-cell secretory function independently of insulin sensitivity in the context of differential metabolic clearance rates of insulin (MCRI) in nonhuman primates (NHPs).

**Methods:**

Insulin secretion rate (ISR) was derived from deconvolution of serial C-peptide concentrations measured during a 5 stage graded glucose infusion (GGI) in 12 nondiabetic (N), 8 prediabetic or dysmetabolic (DYS) and 4 overtly diabetic (DM) cynomolgus monkeys. The characterization of the monkeys was based on the fasting glucose and insulin concentrations, glucose clearance rate measured by intravenous glucose tolerance test, and insulin resistance indices measured in separate experiments. The molar ratio of C-peptide/insulin (C/I) was used as a surrogate index of hepatic MCRI.

**Results:**

Compared to the N monkeys, the DYS with normal glycemia and hyperinsulinemia had significantly higher basal and GGI-induced elevation of insulin and C-peptide concentrations and lower C/I, however, each unit of glucose-stimulated ISR increment was not significantly different from that in the N monkeys. In contrast, the DM monkeys with β-cell failure and hyperglycemia had a depressed GGI-stimulated ISR response and elevated C/I.

**Conclusions:**

The present data demonstrated that in addition to β-cell hypersecretion of insulin, reduced hepatic MCRI may also contribute to the development of hyperinsulinemia in the DYS monkeys. On the other hand, hyperinsulinemia may cause the saturation of hepatic insulin extraction capacity, which in turn reduced MCRI in the DYS monkeys. The differential contribution of ISR and MCRI in causing hyperinsulinemia provides a new insight into the trajectory of β-cell dysfunction in the development of diabetes. The present study was the first to use the GGI and C-peptide deconvolution method to quantify the β-cell function in NHPs.

## Background

Loss of glucose-stimulated insulin secretion from pancreatic islet β-cells is one of the fundamental defects in the pathogenesis of type 2 diabetes (T2D) and portends overt diabetes in humans and in nonhuman primates (NHPs). Understanding of the mechanisms leading to β-cell dysfunction is crucial for discovery and development of novel therapies to prevent and treat diabetes. Historically, rodents have served as the mainstay of *in vivo* preclinical models for studying normal and abnormal β-cell function. However, spontaneous obesity, dysmetabolism (metabolic syndrome) and diabetes are uncommon in rodents and their natural history and pathogenesis is inconsistent with clinical observations in humans. Notably, multiple studies have demonstrated that many of the molecular and histologic characteristics of dysfunctional rodent β-cells deviate from humans, while those from NHPs are highly similar to humans in both islet architecture and β-cell function. For example, amyloid deposits which are derived from islet-associated polypeptide (IAPP) have been frequently observed in the islets in T2D humans [1] and NHPs [2], but are not observed in rodents. Islet studies in NHPs have uncovered considerable overlap with findings in humans. The natural history of T2D along with changes in β-cell function has been best described in *Macaca mulatta*. The NHP models have been characterized for their use in quantifying β-cell function, and are indispensable preclinical translational models for investigating the pathogenesis of T2D and for testing or profiling novel anti-diabetic agents.

About 50% of newly secreted insulin molecules are extracted by the liver in the first pass and the extent of hepatic insulin clearance varies by disease state and insulin load [3]. In contrast, the C-peptide, co-secreted with insulin from the β-cell on an equimolar basis, is not significantly cleared by the liver [4,5]. *In vivo* assessment of dynamic β-cell insulin release is most robustly accomplished in blood samples taken directly from the hepatic portal vein [6], an approach rarely practical in humans, although applied sometimes in NHP studies, because portal vein catheterization requires highly invasive surgery that is not readily available in most research settings. Fortunately, the problem can be addressed by exploiting the co-secretion and differing clearance properties of insulin and C-peptide [7]. Hence, by mathematically modeling (with deconvolution) serially measured circulating C-peptide and insulin concentrations under conditions of β-cell stimulation, a surrogate of pre-hepatic insulin secretion rate (ISR) can be derived [8]. This deconvolution approach represents the most useful noninvasive method of quantifying pre-hepatic insulin secretion and has been used in clinical research [9], but not yet been established, validated and used in an NHP species over a range of different β-cell function conditions, including normal, prediabetes with disturbed metabolism, and overt T2D, to quantify alterations in insulin secretion.

Glucose-stimulated insulin release can be produced by a single glucose injection, such as during an intravenous glucose tolerance test (ivGTT) [10,11], or by a fixed dose of glucose infusion [12,13]. However, these techniques do not allow construction of a dose–response curve between glucose and ISR at various glucose concentrations. The insulin secretory response to a more slowly and physiologically increasing glucose stimulus is used here to uncover novel features of β-cell function [14]. The graded glucose infusion (GGI) has been shown in humans to dose-dependently stimulate the β-cell release of insulin, thus, being considered a method to quantify the β-cell insulin secretory function [15,16]. Therefore, the present study aimed to use the GGI method to quantify β-cell function in response to gradually increaseing blood glucose stimulation, and thus, to investigate the relative contribution of insulin secretion and hepatic insulin clearance in causing hyperinsulinemia in NHPs under different metabolic states. Specifically we have shown that the ISR was increased during fasting conditions in dysmetabolic monkeys, but ISR was not different from normal monkeys in response to glucose stimulation during a GGI, while hepatic insulin extraction differed significantly between the two groups. A major reduction in ISR was observed in the overt T2D monkeys. The present study used the GGI method to quantify the insulin secretory capacity in NHPs ranging from normal to overtly diabetic, and identified major defects in the ISR in the diabetic group and reduced insulin extraction in the prediabetic/dysmetabolic group relative to normals.

## Methods

Twenty four cynomolgus macaques *(Macaca fascicularis)* of both genders were individually housed at Crown Bioscience Inc., Taicang, China in species appropriate cages in a temperature-controlled room maintained at 20 ± 3°C on a 12-hour light–dark cycle. They were fed normal primate chow containing 19% protein, 5% fat and 3.6% fiber (Shanghai Shilin Biotechnology Inc., Shanghai, China) twice daily and had free access to tap water. All of the experimental procedures were approved by the Institutional Animal Care and Use Committee (IACUC) and were performed in accordance with the Guide for the Care and Use of Laboratory Animals (ILAR, National Academies Press, USA 2011). All experimental procedures were carried out under light ketamine anesthesia initiated with 10 mg/kg intramuscularly and supplemented to maintain sedation during each experiment.

### Intravenous glucose tolerance test (ivGTT)

In the absence of well-defined criteria to classify diabetes and glucose intolerance in NHPs, we used both fasting glucose and insulin as well as the glucose clearance rates obtained from ivGTTs performed before the GGI experiments to evaluate the metabolic stages of the monkeys according to an established protocol [17,18]. The glucose solution (50% dextrose, 0.25 g/kg) was administered intravenously in a single bolus over a 30 second period. Venous blood samples were obtained immediately before and at 3, 5, 10, 15, 20 and 30 min after glucose administration. Glucose disappearance or clearance rate (Kglucose) was expressed as the rate of glucose being removed from the circulation with a one compartment kinetic model calculated by the slope of a linear trend best matching the natural logarithm of the blood glucose concentrations at each time point, by fitting a straight line using the least squares method with Microsoft Excel formula: LINEST (LN (blood glucose concentrations at each time point), 3, 5, 7, 10, 15, 20, 30 min).

The monkey colony was objectively divided into 3 distinct groups based on below criteria: metabolically normal (N), prediabetic or dysmetabolic (DYS) and overly diabetic (DM) with fasting glucose < 60, 60–100 and > 100 mg/dL, and/or Kglucose > 2, 2–1.5 and < 1.5 g/min, respectively. The quantitative insulin sensitivity check index (QUICKI) to estimate insulin sensitivity was calculated as 1/[log (fasting blood glucose concentration, mM) + log (fasting serum insulin concentration, mIU/L)] [19-21]. Homeostatic model assessment of insulin resistance (HOMA-IR) was obtained by the fasting glucose-insulin product: fasting blood glucose concentration (mM) x fasting serum insulin concentration (mIU/L) / 22.5 [19,21].

All animals were weight stable prior to study initiation. The monkeys that were classified as DM were recently diagnosed early diabetic, not yet receiving insulin therapy, and were weight stable and negative for urinary ketones. Preliminary analyses using multiple regression analysis indicated that sex did not significantly contribute to variation in any of the GGI and ivGTT outcome variables and was subsequently not further considered in comparisons between the groups [19,21].

### Graded glucose infusion (GGI)

#### Experimental procedures

Under ketamine anesthesia as described above, intravenous catheters were placed in the cephalic and saphenous veins. Glucose (20% dextrose) was continuously infused into the saphenous vein over five 40-min stages starting at 2 mg/kg/min and doubling at each successive stage up to 32 mg/kg/min. Blood was sampled at baseline (−10 and −3 min before the start of glucose infusion) and serially thereafter every 20 min for 200 min. Samples were placed on ice, centrifuged within 30 min of collection and serum obtained for biochemical assays.

Insulin secretion rate (ISR): Peripheral C-peptide levels can be used to estimate pre-hepatic insulin secretion. Under non-steady state conditions, however, C-peptide kinetics are non-linear and subject to deconvolution. Thus, C-peptide secretion, thereby pre-hepatic insulin secretion patterns can be reassembled with the Regularization Method by deconvolution of peripheral C-peptide kinetics, using a two-compartment model implemented in the software program ISEC [8]. This program was developed to estimate insulin secretion and standard parameters for C-peptide clearance based on age, sex, weight, length (height), and metabolic status in humans.

Hepatic metabolic clearance rate of insulin (MCRI): Insulin and C-peptide are co-secreted from the β-cell on an equimolar basis [4,5]. Both *in vivo* and *in vitro* reports have demonstrated a large capacity of the liver to extract insulin from the portal blood [3-5], but little or no C-peptide removal occurs across the liver [22]. Thus, a surrogate hepatic insulin extraction or clearance rate can be assessed noninvasively from the C-peptide/insulin molar ratio (C/I) measured in the peripheral blood [6].

### Analyses

Blood glucose concentration was measured onsite during the experiment with a glucometer (Accu-Chek® Performa). The same blood samples were taken for measurement of serum glucose concentrations by the glucose oxidase method after the experiment. The glucose concentration values obtained by the two different methods from blood or serum were highly correlated (r=0.99, p<0.001). Thus, the blood glucose concentrations were used for the data analysis in this manuscript. Serum insulin and C-peptide levels were determined with an electrochemiluminescence immunoassay by a clinical instrument (Roche, Cobas 601).

### Data and statistical analyses

Data are presented as mean and standard error of the mean. The time course (glucose infusion rate) - response (blood glucose, insulin, C-peptide, C/I and ISR) curves were constructed. Comparison of the parameters at each time point after glucose infusion with the baseline value at time 0 min before glucose infusion was carried out by paired t test. Serial data over the time course were integrated into area under the curve (AUC) by the standard trapezoid method as the average response over the entire GGI period. The β-cell insulin secretory function in response to graded glucose stimulation was analyzed by construction of the logarithmic blood glucose concentration-response curves for insulin, C-peptide, C/I and ISR, respectively to quantify each unit of blood glucose change-induced unit of change in the above parameters. The correlation coefficients were analyzed with a formula in Microsoft Excel: CORREL (series of the measured parameters, LN (series of blood glucose concentration)). The sensitivity of glucose-stimulated insulin release was then quantified by the slopes of the logarithmic blood glucose concentration-response curves by best fitting a straight line on these parameters against the natural logarithm of blood glucose concentrations. The least squares method was used with a formula in Microsoft Excel: LINEST (the measured parameters against LN (each blood glucose concentration)). Group means were compared using ANOVA with Fisher LSD pairwise *post hoc* comparisons. Statistical significance was set at p<0.05. The data were analyzed using the XLSTAT 2012 (Addinsoft Inc.) in MS Excel 2010 (Microsoft Inc.).

## Results

### Basal characteristics at fasting condition

**Figure 1:**
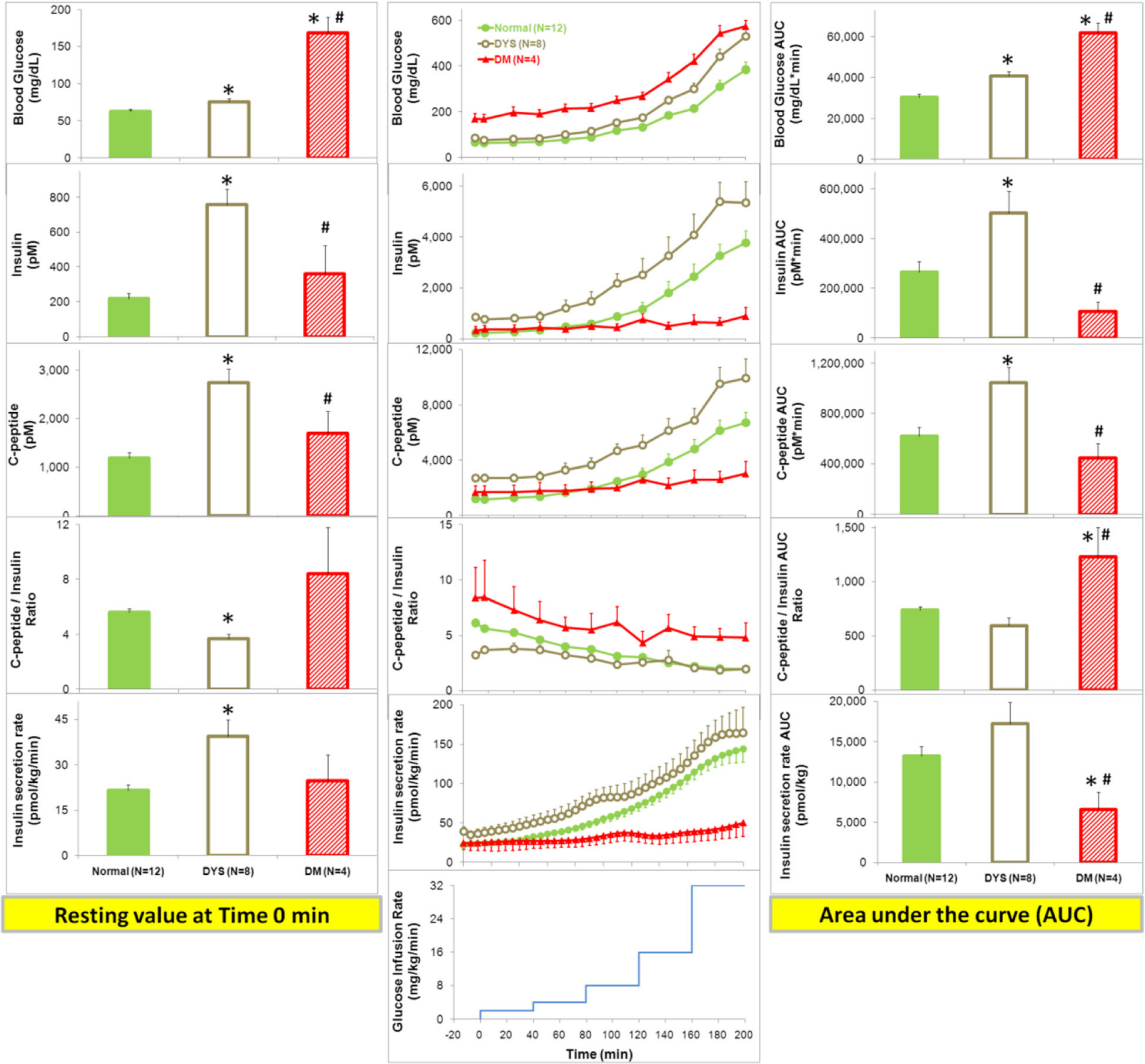
**Time course of the blood glucose (Top), serum concentrations of insulin (Second) and C-peptide (Third), molar ratio of C-peptide / insulin (Fourth), and calculated insulin secretion rate (ISR) by deconvolution of serum C-peptide concentration decay curve (Fifth) induced by graded glucose infusion (bottom) in the normal (N), dysmetabolic (DYS) and diabetic (DM) cynomolgus monkeys. Left**: Basal level at the resting condition before the graded glucose infusion. **Middle**: Time course during the graded glucose infusion. **Right**: Area under the curve (AUC) over the entire 200 min time course. *Significantly different from normal group, ^#^ significantly different from dysmetabolic group, p<0.05, by ANOVA with Fisher (LSD) *post hoc* comparisons.

Using the overnight fasting glucose and insulin, as well as the Kglucose value obtained from the ivGTT to separate the metabolic states of the NHP colony, the fasting serum glucose concentration was significantly higher in the DSY, and, by definition, the highest in the DM group. The Kglucose was significantly lower in the DYS, and the lowest in the DM group, compared to the N group (Table 1). The insulin sensitivity index QUICKI was significantly lower in both the DYS and DM groups compared to the N group, while the insulin resistance index HOMA-IR was greater in the DYS and DM groups relative to the N group. There were no differences in either QUICK or HOMA-IR between the DYS and DM groups. The DM and DYS NHPs were significantly older than the N group, however, the body weights were not significantly different among the groups. Since there were proportionally more male monkeys in the N group, this sex difference unequally contributed to the average body weight; thus the DM and DYS were obese relative to the N monkeys. Using the baseline value before the GGI (Figure 1, left), the fasting blood glucose concentrations were slightly, but significantly higher in the DYS and the highest in the DM compared to the N group, however, the fasting insulin and C-peptide, as well as ISR, were significantly higher in the DYS than in either the N or the DM groups. The basal C/I molar ratio was significantly lower in the DYS, and tended to be higher but was not statistically significantly elevated in the DM (probably due to the great variability) compared to the N group.

**Table 1:** Descriptive characteristics (mean ± standard error)

	**Normal (N)**	**Dysmetabolic (DYS)**	**Diabetic (DM)**
**Objects (Male/Female)**	12 (11/1)	8 (4/4)	4 (2/2)
**Age (years)**	9.7 ±1.1	16.0 ± 1.1*	16.1 ± 1.2*
**Body Weight (kg)**	8.7 ± 0.8	9.0 ± 1.2	9.0 ± 1.8
**Fasting Serum Glucose (mg/dL)**	43.1 ± 1.8	68.5 ± 5.0*	171.5 ± 22.3*#
**Kglucose (g/L/min)**	2.5 ± 0.2	1.9 ± 0.2*	1.3 ± 0.1*#
**QUICKI (1/mM+mIU/L)**	0.55 ± 0.02	0.38 ± 0.01*	0.43 ± 0.05*
**HOMA-IR (mM x mIU/L)**	3.6 ± 0.6	21 ± 2.2*	23 ± 11*

* or #: significantly different from N or DYS group, respectively, p<0.05, by ANOVA with Fisher (LSD) *post hoc* comparisons.

### GGI-stimulated insulin secretion

The GGI procedure resulted in a progressive rise in blood glucose concentrations in all groups (Figure 1, middle), however, the overall glucose responses shown as AUCs were significantly greater in the DYS and the greatest in the DM compared to the N monkeys (Figure 1, right). The gradual elevation in blood glucose concentrations increased the ISR, thereby increasing the serum concentrations of insulin and C-peptide to a greater degree in the DYS than N NHPs, but the changes were very minimal in the DM group. The differences were more dramatic in the late phase of the GGI time course curves (Figure 1, middle), as well as in the integrated AUCs representing average change during the entire GGI period (Figure 1, right). There was a gradual decline in the C/I in all 3 groups, however, the change was minimal in the DYS group. Thus, the C/I time course curves gradually merged between the DYS and N groups with no difference in AUC integrated over 200 min, although the ratio remained significantly higher in the DM group. At the lowest glucose infusion rate (2 mg/kg/min), all of the above responses to GGI were minimal with no statistical significance compared to the basal levels at time 0 min before glucose infusion, however, the GGI-induced responses were fully saturated at 32 mg/kg/min (Table 2). Except for the increase in blood glucose concentration, none of the other responses (insulin, C-peptide, C/I and ISR) to GGI reached statistical significance at any time points relative to the baseline values in the DM group.

**Table 2:** The first time point (min) when the infusion of glucose-induced responses reached a statistically significant difference from the respective baseline values at time 0 min measured by paired t test, p<0.05

**(min)**	**Normal**	**DYS**	**DM**
**Glucose**	60	60	80
**Insulin**	40	100	NS
**C-Peptide**	40	100	NS
**C/I**	40	80	NS
**ISR**	20	75	NS

### Sensitivity of β-cell insulin secretion in response to circulating glucose stimulation

**Figure 2:**
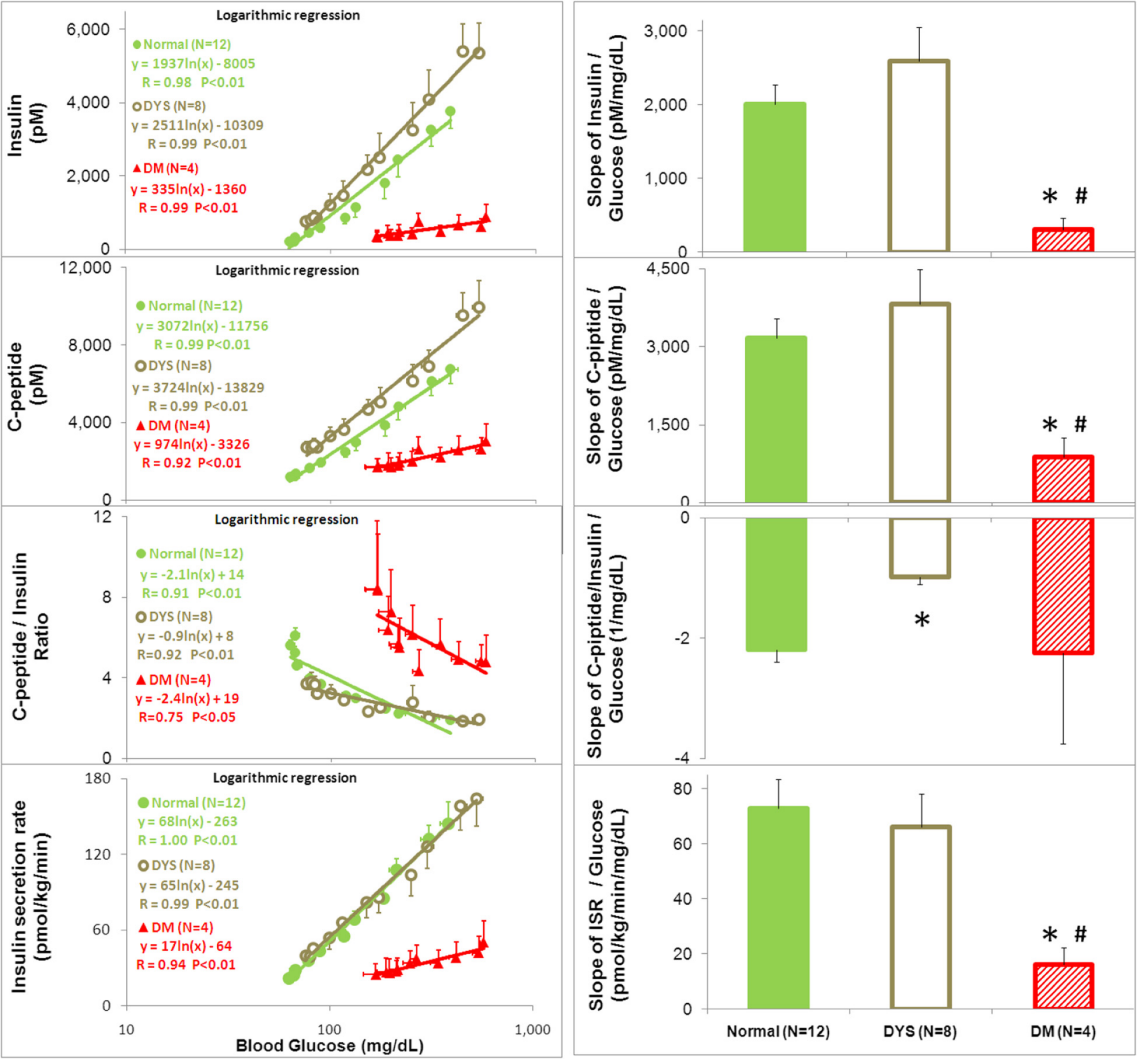
**Blood glucose concentration-response curves (Left) for insulin (top), C-peptide (Second), molar ratio of C-peptide / insulin (Third), insulin secretion rate (bottom) and the slopes of the concentration response curves (Right) in the normal (N), dysmetabolic (DYS) and diabetic (DM) cynomolgus monkeys.** *Significantly different from normal group, ^#^ significantly different from dysmetabolic group, p<0.05, by ANOVA with Fisher (LSD) *post hoc* comparisons.

The β-cell insulin secretory function in response to glucose stimulation was also analyzed by the blood glucose concentration-response curves for insulin, C-peptide, and ISR (Figure 2, left) and the slopes of these curves were used to measure the sensitivity of β-cell in response to the unit increment of blood glucose concentration (Figure 2, right). The responses of all of the parameters, including insulin, C-peptide and ISR, were highly and positively correlated with the blood glucose concentrations (Figure 2, Left). The glucose concentration - response curves for insulin and C-peptide were left-shifted with a slight, but not significant increase in the slope in the DYS, and dramatically right-shifted with a significantly diminished slope in the DM, relative to the N group. The glucose concentration-response curves for ISR in the N and DYS groups did not differ from each other, but this relationship was minimal in the DM group, indicating that the β-cell insulin secretory response to glucose stimulation was compromised in the DM NHPs.

In contrast, the C/I response curves were negatively correlated to the rising blood glucose concentrations in all 3 groups, indicating that gradual elevation of glucose concentration over the time course during the GGI was associated with progressively reduced hepatic insulin extraction, thus, considered an impairment of the MCRI. The curves between the N and DYS were close together, but had a right shift in the DM group (Figure 2, left). The slopes of glucose concentration - response for C/I curves were similar between the N and DM, but significantly lower in the DYS group (Figure 2, right), indicating a higher level of saturation of the hepatic insulin extraction capacity probably due to the hyperinsulinemia.

## Discussion

The present study examined the changes in β-cell insulin secretory function during progressive stimulation by rises in glucose levels with a GGI procedure in the cynomolgus monkeys ranging from normal to overtly diabetic. Relative to the normal monkeys, the DYS monkeys, under fasting conditions, were characterized by a slight, but significant hyperglycemia, accompanied by a marked hyperinsulinemia and enhanced ISR with a lower systemic glucose clearance and reduced hepatic MCRI. In contrast, the DM monkeys had a marked hyperglycemia and a further diminished systemic glucose clearance, but a higher hepatic MCRI. During the GGI, there were significantly enhanced insulin and C-peptide responses in the DYS compared to the N group, which was dramatically depressed in the DM group. However, the GGI-induced increase in ISR was not significantly different between N and DYS, although markedly diminished in the DM monkeys.

One hypothesis is that the pancreatic β-cells can maintain normal glucose homeostasis in the insulin-resistant state during the prediabetic or dysmetabolic phase by a compensatory increase in the β-cell response to glucose stimulation [23]. Diabetes develops when there is deterioration in β-cell insulin secretory responsiveness to glucose due to β-cell failure [24]. Similar to dysmetabolic humans, the DYS monkeys in the present study also showed marked hyperinsulinemia, a characterization of increased insulin resistance as defined by HOMA-IR or decreased insulin insensitivity as defined by QUICKI. Using the stepwise increment in glucose infusion rate to gradually elevate blood glucose concentrations to stimulate β-cell insulin secretion, quantified as ISR, these DYS monkeys were determined to have preserved β-cell insulin secretory function, fully capable of increasing ISR to compensate for the reduced tissue sensitivity to insulin, and were thus, capable of maintaining glucose homeostasis. In contrast, the DM monkeys’ β-cells were severely impaired, and incapable of increasing fasting as well as GGI-stimulated ISR in the face of severe insulin resistance, thus, leading to hyperglycemia. These data clearly demonstrate that the β-cell function was enhanced in the fasting state and maintained during the GGI in the DYS, but significantly depressed in the DM monkeys. These results are consistent with the reports in insulin-resistant patients whose glucose homeostasis can be maintained when the defect in insulin action is adequately compensated for by an increase in insulin concentration.

While hyperinsulinemia has been generally attributed to β-cell hypersecretion of insulin, it is not clear whether it is a primary event governed either by central neural or β-cell intrinsic regulation, or by a secondary event arising from an adaptive response to diminished peripheral insulin sensitivity in the dysmetabolic state [25]. It has been previously shown that non-diabetic insulin resistant individuals have a left-shift of the glucose-stimulated ISR dose–response curve when compared with normal individuals; thus, at any given glucose concentration, insulin-resistant individuals secrete more insulin than insulin-sensitive ones [26]. Furthermore, there is evidence that the improvement in insulin sensitivity associated with weight loss [10-13] or with 3 months of rosiglitazone treatment [15] right-shifted the glucose-stimulated ISR dose–response curve toward normal, leading to less insulin secretion at any given glucose concentration. Several factors have been hypothesized to contribute to the progressive loss of β-cell function, including glucotoxicity and lipotoxicity, amyloid accumulation, an increase in pro-inflammatory cytokines, and increased secretory demand because of peripheral insulin resistance [2,27]. The increase in insulin requirement driven by insulin resistance may play a contributory role in the deterioration of β-cell function [27]. Therefore, interventions that help to preserve or improve β-cell function and/or ameliorate insulin resistance are likely to be effective in preventing or treating diabetes [28]. Techniques to robustly quantify these improvements *in vivo* in clinically relevant animal models are essential to the evaluation of potential clinical efficacy in the discovery and development of novel anti-diabetic therapeutics.

Blood insulin concentration is a dynamic balance between insulin production/secretion and clearance. In addition to the changes in β-cell ISR, the MCRI is another contributing factor to circulating insulin levels [26,29]. Using the C/I ratio as an index of hepatic insulin extraction rate or MCRI, the present data demonstrate that the basal C/I was significantly lower, which may also contribute to hyperinsulinemia in the DYS cynomolgus monkeys. In contrast, the average C/I during GGI calculated by AUC was significantly higher in the DM group, which could contribute to the low circulating insulin levels during the GGI. Indeed in insulin-resistant individuals, MCRI has been shown to be decreased (26), suggesting that changes in insulin resistance can independently impact insulin clearance (15). The exact mechanism of such differences in hepatic insulin extraction rate in different metabolic states is still unclear. The liver is a major organ that clears insulin directly secreted from the β-cells during the first pass through the portal circulation with limited capacity. Previous studies have suggested that hepatic insulin extraction rate may decline due to saturation of hepatic insulin uptake at higher concentrations [22]. Indeed, Hansen et al. reported that in dysmetabolic rhesus monkeys, increasing portal insulin concentration above 1,000 pM leads to a decrease of hepatic insulin extraction by 40 to 60% [6]. This decline, therefore, contributes approximately 50% to the overall development of hyperinsulinemia, the remainder of the increase being related to β-cell hypersecretion of insulin. Using a double-label method to measure MCRI and hepatic extraction of exogenous insulin in conscious dogs, Pye et al. also reported that as portal insulin levels were increased from basal level to 3,738 pM, hepatic insulin uptake decreased from 61% to 29%, while the MCRI declined 3 fold [30]. These data are in accord with the reports that a saturation of hepatic insulin extraction occurs at elevated insulin concentrations approaching 1,800 pM in anesthetized dogs [31], 1,804 - 1,955 pM in dysmetabolic rhesus monkeys [6], and 1,680 pM in men during exogenous insulin infusion [32]. These levels are consistent with the range of insulin concentrations in the present study during GGI. Thus, the present data provide new evidence in the cynomolgus monkey model of dysmetabolism and diabetes that in addition to the β-cell insulin hypersecretion, reduction in hepatic insulin clearance possibly due to a saturation mechanism also contributes to the development of hyperinsulinemia.

Concerning the GGI protocol itself, the present data have shown that the effects at 2 mg/kg/min glucose infusion rate were so minimal in all groups that this low infusion rate can be eliminated as non-informative for future studies of β-cell secretion/function. At the other end of the infusion rates tested here, the highest rate of 32 mg/kg/min used in the present study produced extraordinarily high glucose concentrations at non physiological levels (often over 400 mg/dL) and saturated insulin responses, which provided no additional information over that provided by the 16 mg/kg/min infusion rate, indicating that this high level can also be eliminated from future GGI protocols. Thus, the most cost and effort effective, and scientifically useful glucose infusion rates for both humans and NHPs appear to be at the 8 and 16 mg/kg/min (40 min. each rate), as suggested in a study by Ehrmann et al. and this allows ISR to be examined over glucose levels at a more physiological range from 140–250 mg/dL in normal humans [14].

One limitation of the present study is the use of C-peptide clearance parameters estimated from a human population [8]. To date, C-peptide deconvolution approaches to quantify β-cell function under stimulated conditions have not been described in an NHP species. Direct measurement of C-peptide kinetics individually in each monkey would be laborious and time consuming. Although there are other more sophisticated mathematical models to analyze the glucose-insulin metabolism and insulin sensitivity, in the present study, we adopted the most simple methods and indices commonly used in the literature.

## Conclusion

The present data revealed that both an increased ISR and reduced hepatic MCRI at the fasting stage contribute to the hyperinsulinemia in the DYS monkeys. However, there was no significant difference in glucose-stimulated increase in ISR between the N and DYS groups and a significantly reduced MCRI during GGI in the DYS monkeys, indicating that the exaggerated elevation of insulin and C-peptide concentrations during GGI is predominantly contributed by a diminished hepatic insulin clearance, probably a result of hyperinsulinemia-induced saturation of the hepatic insulin extraction capacity. In diabetic NHPs with impaired β-cell function, the GGI-induced ISR was greatly depressed with a higher MCRI, thus hyperglycemia developed. Therefore, the GGI method combined with analysis of ISR by C-peptide deconvolution in the NHP model of diabetes and metabolic disorders provides a powerful translational tool for insight into the trajectory of β-cell dysfunction in the development of diabetes and for preclinical profiling of efficacy of novel pharmaceutical agents targeting improvements of β-cell function.

## Abbreviations

GGI: Graded glucose infusion; ivGTT: Intravenous glucose tolerance test; N: Normal or nondiabetic; DYS: Dysmetabolic or prediabetic; DM: Type 2 diabetic; Kglucose: Glucose disappearance rate; ISR: Insulin secretion rate; C/I: Molar ratio of C-peptide/insulin; MCRI: Metabolic clearance rate of insulin; AUC: Area under the curve; T2D: Type 2 diabetes; QUICKI: Quantitative insulin sensitivity check index; HOMA-IR: Homeostatic model assessment for insulin resistance.

## Competing interest

All the authors are the employees of Crown Biosciences, Inc, except for BCH who is a scientific advisor of Crown Bioscience, Inc. There is no potential conflict of interest relevant to this article.

## Authors’ contributions

YXW took charge of the study design, results interpretation and manuscript writing, has full access to all the data in the study and is responsible for the integrity and accuracy of the data analysis; XLW was involved in performing the experiments, data analysis and manuscript writing; BCH was involved in the analysis and interpretation of the data and in discussion and manuscript writing; DS contributed to the data analysis; YPF, FLD and BDW performed the experiments; YXC and FMG contributed to discussions. All authors read and approved the final manuscript.

## Authors’ information

Dr. Yixin (Jim) Wang is the president of the International Institute of Biomedical Research, a Crown Bioscience Company, operating in Kannapolis, NC USA and the senior vice president, head of the cardiovascular metabolic diseases research of Crown Bioscience (Taicang) Inc. operating in Taicang, China where this study was performed. He is in charge of the research and operations in both the USA and China facilities.
